# Supplementary material for: Vaccine Hyporesponse Induced by Individual Antibiotic Treatment in Mice and Non-Human Primates Is Diminished upon Recovery of the Gut Microbiome
Source: Vaccines (Basel). 2021 Nov 17;9(11):1340. doi: 10.3390/vaccines9111340 (PMC8619314; doi:10.3390/vaccines9111340)
Supplement: Supplementary file 1 [file vaccines-09-01340-s001.zip › vaccines-1383768-supplementary.pdf]

Supplemental Figures

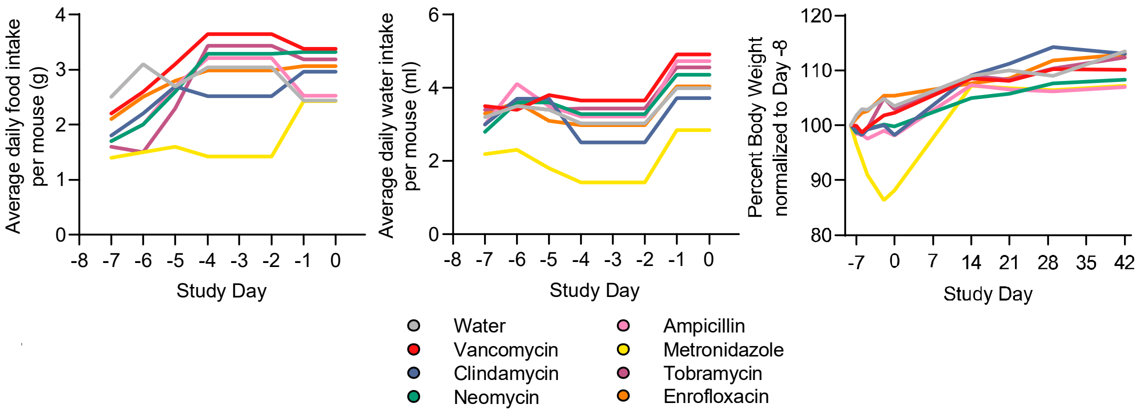

Figure S1. Antibiotic treatment does not affect health measures in BALB/c mice. Food intake, water intake, and body weight normalized to that at the beginning of the study [100%] shown in Figure 1.

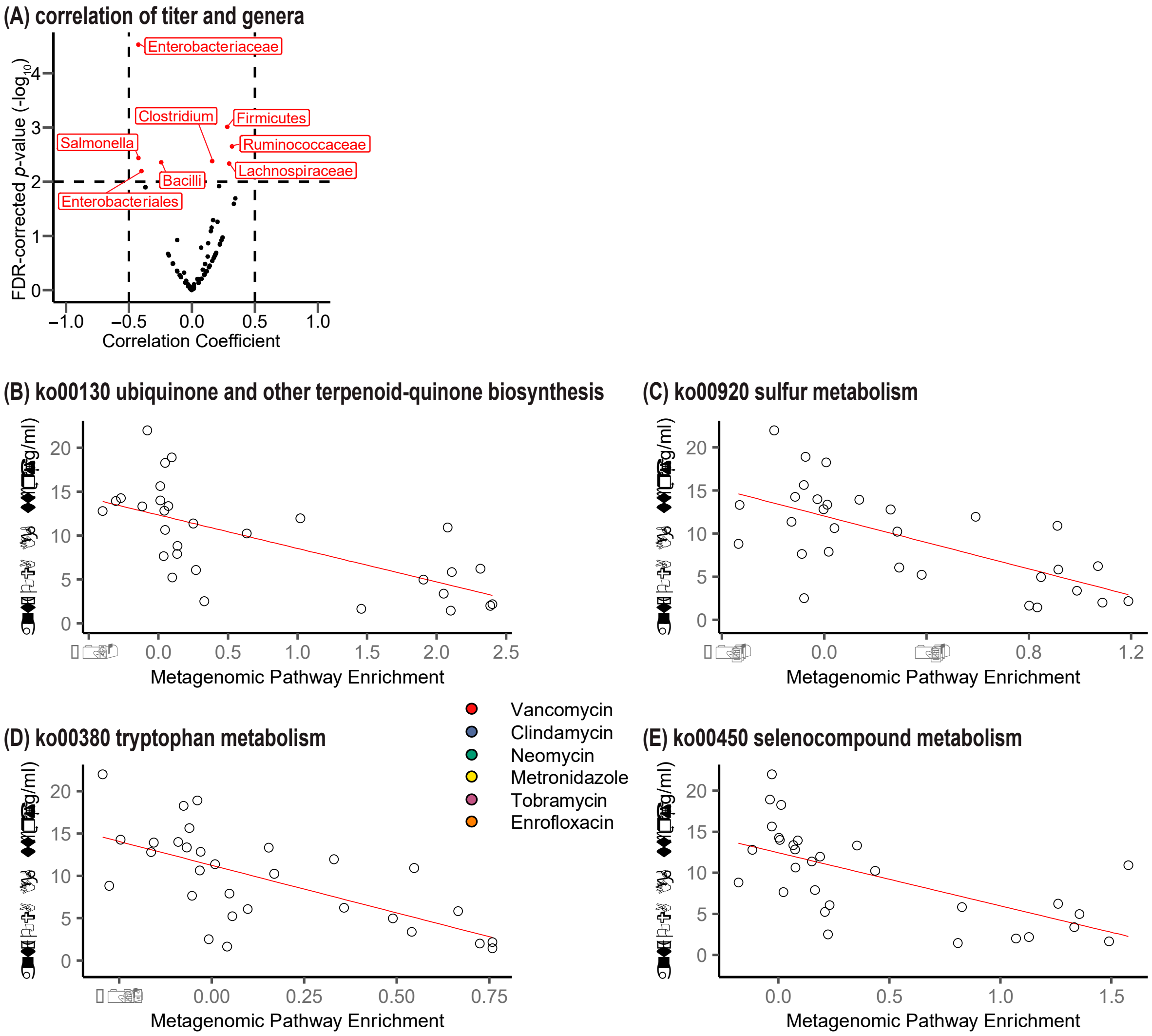

Figure S2. Anti-ova IgG titers do not strongly correlate with abundances of bacterial genera across antibiotic treatments or enrichment of metagenomic pathways. (A) Volcano plot showing Spearman correlation analysis (non-parametric test used to accommodate microbiome data qualities) of anti-OVA IgG titers and abundances of specific bacterial genera at Day 0. Red dots represent taxa that were statistically significantly correlated (FDR-corrected p-value < 0.01), although the correlation coefficient did not exceed 0.5, indicating negligible relevance. (B-E) Correlation scatter plots of each individual mouse's OVA-specific IgG titer and metagenomic enrichment of the indicated Kegg orthology pathways relative to water-treated animals. Ampicillin-treated animals were not included in the metagenomic analysis due to poor metagenomic sequence yield. Red line indicates linear regression; gray shading represents 95% confidence interval. Pearson correlation, FDR-value < 0.005, r < -0.60).

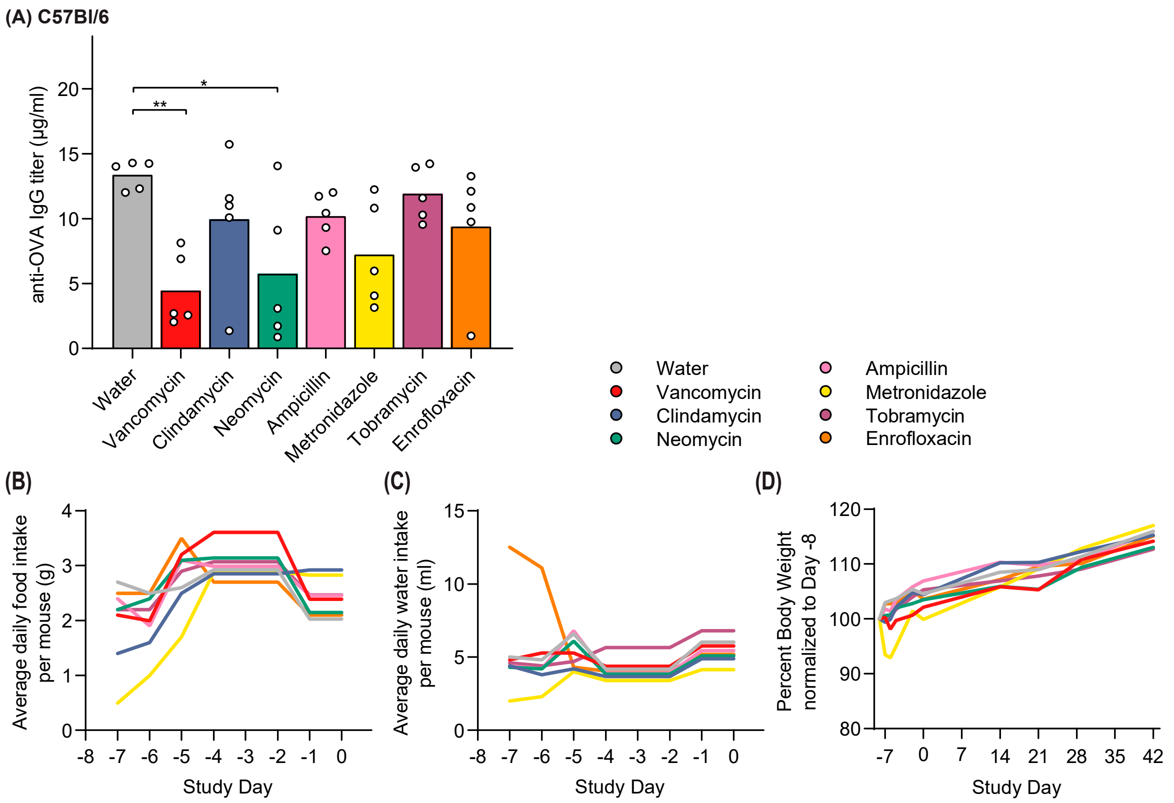

Figure S3. Antibiotic treatment disrupts antibody response to vaccination but does not affect health measures in C57Bl/6 mice. C57Bl/6 mice were treated as described in Figure 1. (A) Serum OVA-specific IgG titers were measured at Day 42 after immunization. Measurements from individual mice (circles) are superimposed on group mean (line). * $p < 0.05$, ** $p < 0.01$ compared to water by Dunnett's test. (B-D) Food intake, water intake, and body weight normalized to that at the beginning of the study [100%].

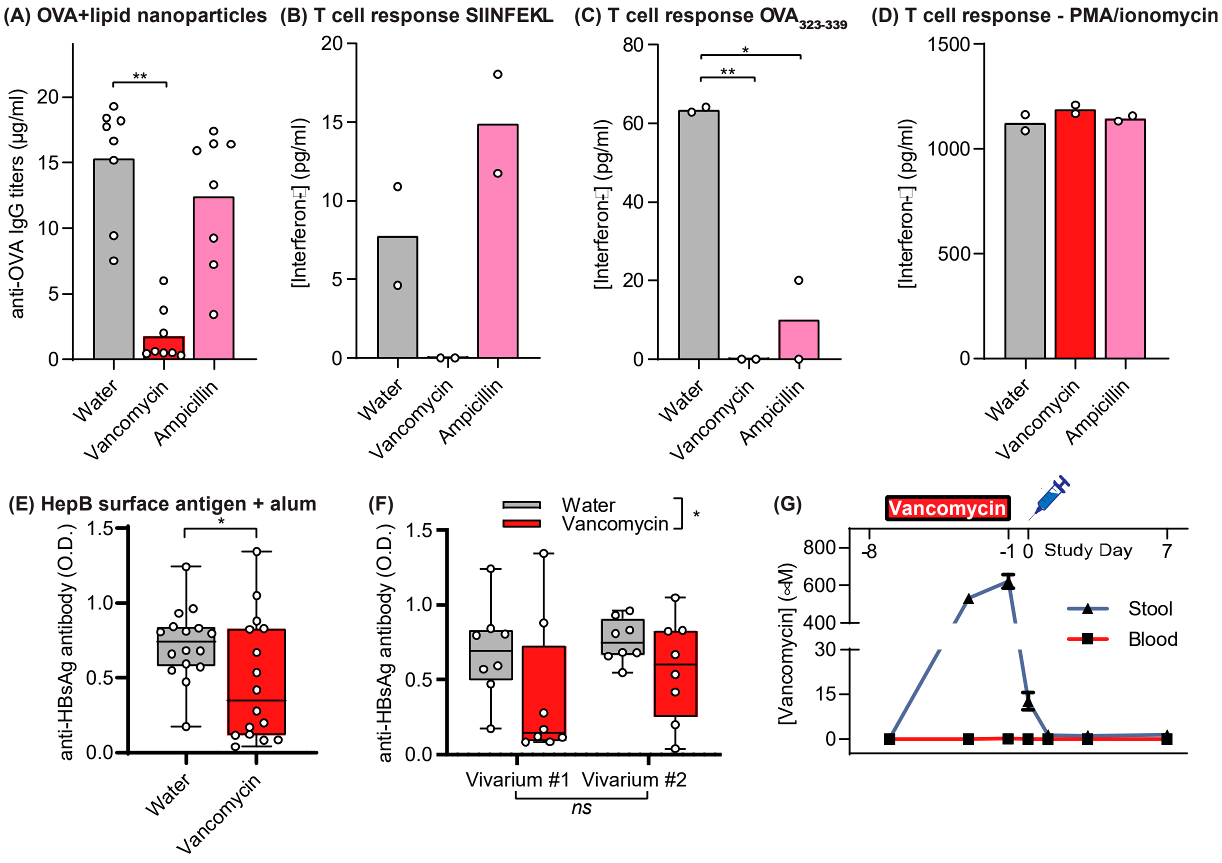

Figure S4. Vancomycin treatment affects response to vaccines with different adjuvants and antigens. (A-D) BALB/c mice were treated with ampicillin or vancomycin for 7 days, followed by one day without antibiotic treatment before immunization with ovalbumin and VA-510 LNPs at Days 0 and 21. (A) Quantification of OVA-specific IgG titers at Day 41. (B-D) Interferon-gamma production by splenocytes from antibiotic-treated mice in response to stimulation with the indicated molecules. (E-G) Separate cohorts of BALB/c mice at vivaria in different states were treated with vancomycin for 7 days, followed by one day without antibiotic treatment before immunization with HBsAg and alum at Days 0 and 21. Serum HBsAg-specific IgG was quantified at Day 35 and plotted across both cohorts (E) or separated by cohorts (F). Measurements from individual mice (circles) are superimposed on group mean (line). Boxplots indicate upper and lower quartiles. (G) Concentrations of vancomycin in mouse stool and blood 7 days before and after the first immunization; data are mean + SEM of 5 mice. * $p < 0.05$, ** $p < 0.01$ compared to water by one-way ANOVA with Dunnett's post-test (A-D), one-sample t test (E), or two-way ANOVA (F).

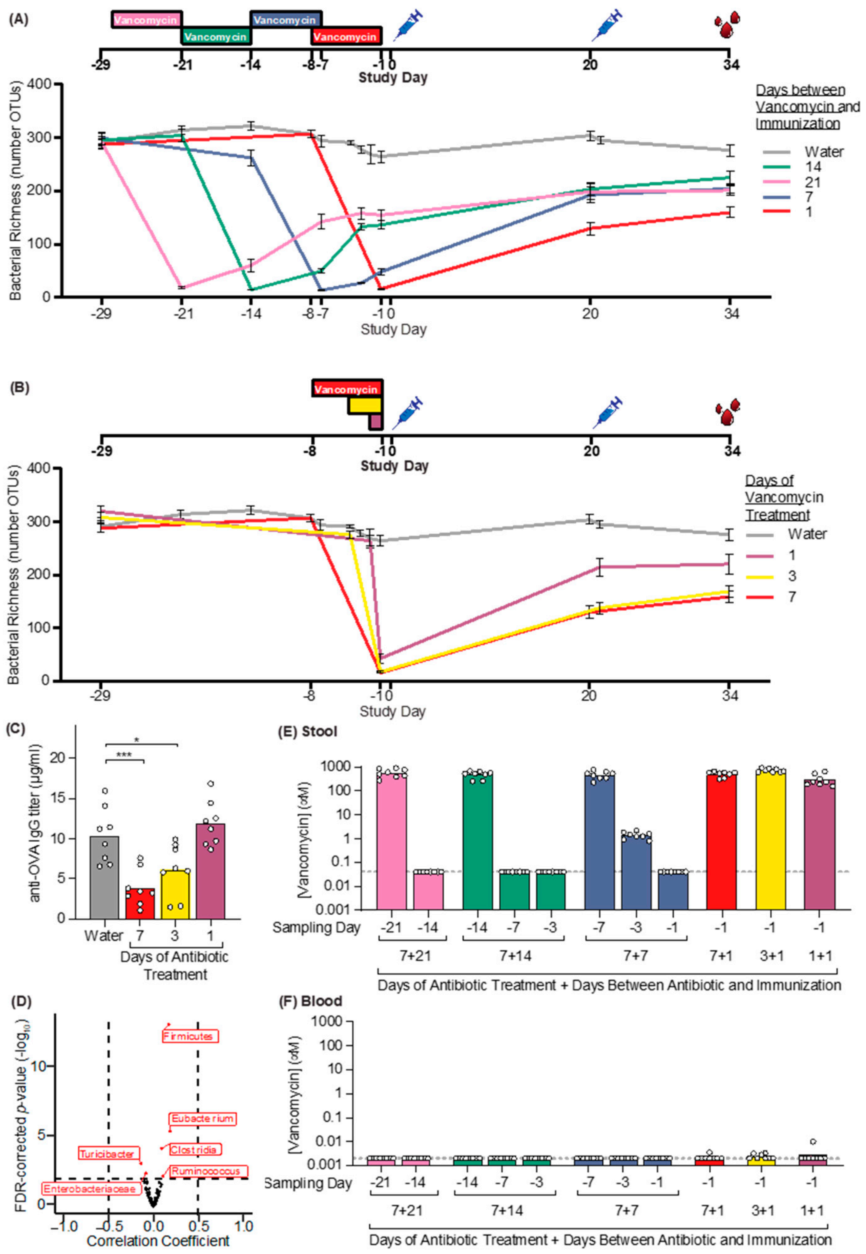

Figure S5. Microbiome recovery after antibiotic treatment. (A) OTU richness (alpha diversity) of groups described in Figure 2. (B,C) In parallel to the samples described in Figure 3, mice were orally treated with vancomycin for 7, 3, or 1 day (rectanges), followed by 1 day without antibiotic treatment, prior to immunization as in Figure 3. Data from the group receiving 7 days vancomycin and 1 day without antibiotic is repeated from Figure 3. (B) OTU richness (alpha diversity) during the study. (C) Serum anti-OVA IgG was measured at Day 34; * $p < 0.05$, *** $p < 0.001$ compared to water by Dunnett's test. (D) Volcano plot showing correlation analysis of anti-OVA IgG titers and abundances of specific bacterial genera at Day -1. Six taxa (red dots) were statistically significantly correlated (FDR-corrected p-value < 0.01; horizontal dashed line), although the correlation coefficient did not exceed 0.5 (vertical dashed lines), indicating negligible relevance. (E,F) Concentrations of vancomycin in mouse stool and blood. Dashed line represents limit of quantification. Measurements from individual mice (circles) are superimposed on group mean (line).

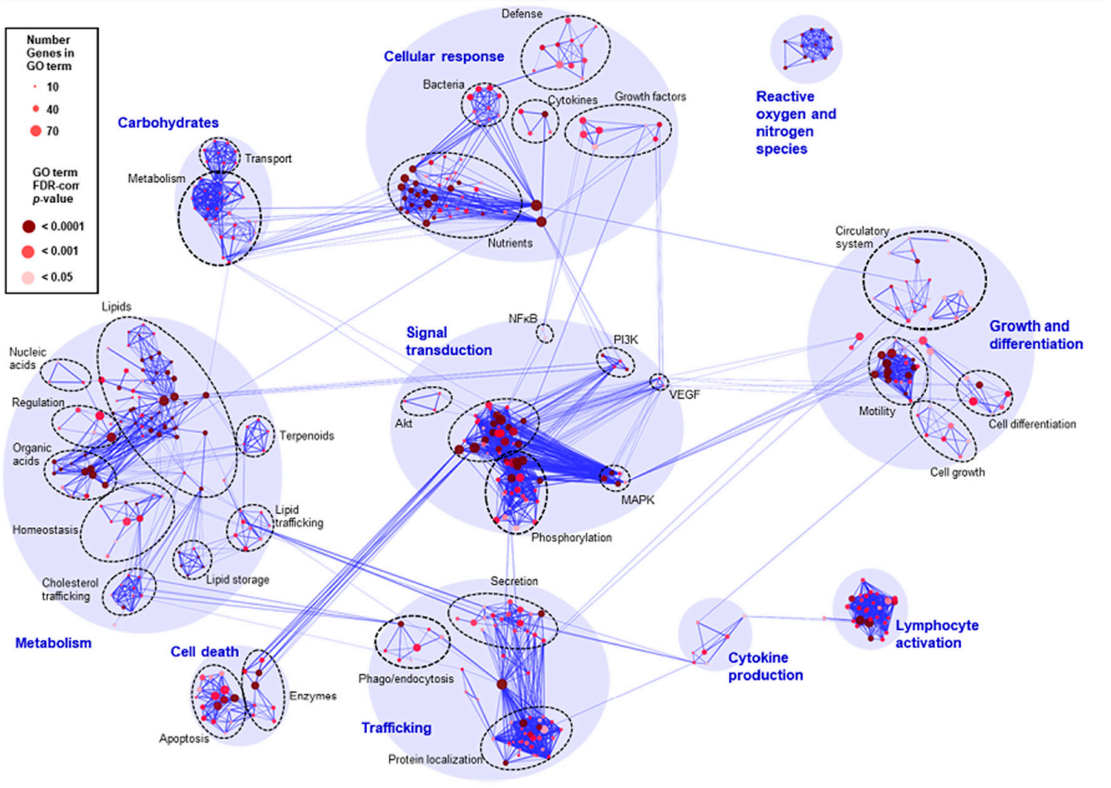

Figure S6. Clustering of enriched GO terms in RNA sequencing of mesenteric lymph nodes from vancomycin-treated mice. Gene set enrichment analysis was used to identify GO terms enriched in the vancomycin-treated mesenteric lymph nodes RNA-sequencing dataset. All enriched GO terms were clustered based on included differentially expressed genes. Node size and color respectively denote the number of genes present in and the statistical significance of enrichment for that GO term; thickness of blue lines represents the similarity between nodes. The following clusters were denoted as "immune-related," subsampled and clustered (Figure 4A), and genes within were used for correlation analysis with OTU abundances (Figure 4B): Lymphocyte activation, Cytokine production, NFκB (Signal Transduction), Cellular Response, and Reactive oxygen and nitrogen species.

Supplemental Tables

	statmean	q.val	KOID	treatment
1	−2.377896	1.130830e−04	ko00511 Other glycan degradation	Baytril
2	−1.833744	3.621323e−03	ko04142 Lysosome	Baytril
3	−2.461681	1.296014e−05	ko00970 Aminoacyl−tRNA biosynthesis	Clindamycin
4	−1.924595	4.323888e−03	ko00970 Aminoacyl−tRNA biosynthesis	Metronidazole
5	−3.116655	3.904034e−09	ko00970 Aminoacyl−tRNA biosynthesis	Neomycin
6	−2.804875	2.016370e−07	ko00970 Aminoacyl−tRNA biosynthesis	Tobramycin
7	−2.613916	2.015826e−06	ko00970 Aminoacyl−tRNA biosynthesis	Vancomycin
8	−1.723265	8.585391e−03	ko04151 PI3K−Akt signaling pathway	Vancomycin

Table S1. Metagenomic KEGG pathways depleted in antibiotics groups, compared to the water control. Pathways shown have FDR corrected p-values < 0.01.

	stat.mean	q.val	KOID	treatment
1	2.583535	4.259424e−06	ko00970 Aminoacyl−tRNA biosynthesis	Baytril
2	1.778945	9.403102e−03	ko00040 Pentose and glucuronate interconversions	Clindamycin
3	1.956431	3.955527e−03	ko00511 Other glycan degradation	Tobramycin
4	2.208632	8.705120e−05	ko02010 ABC transporters	Vancomycin
5	2.210136	1.030133e−04	ko00130 Ubiquinone and other terpenoid−quinone biosynthesis	Vancomycin
6	1.934026	3.585939e−04	ko02024 Quorum sensing	Vancomycin
7	2.016123	3.585939e−04	ko00780 Biotin metabolism	Vancomycin
8	1.930946	3.585939e−04	ko00540 Lipopolysaccharide biosynthesis	Vancomycin
9	1.897841	3.585939e−04	ko01230 Biosynthesis of amino acids	Vancomycin
10	1.908200	3.585939e−04	ko02060 Phosphotransferase system (PTS)	Vancomycin
11	1.813439	1.029094e−03	ko00053 Ascorbate and aldarate metabolism	Vancomycin
12	1.719883	2.587855e−03	ko04122 Sulfur relay system	Vancomycin
13	1.632562	3.462776e−03	ko00790 Folate biosynthesis	Vancomycin
14	1.534577	6.717780e−03	ko00400 Phenylalanine, tyrosine and tryptophan biosynthesis	Vancomycin
15	1.504306	8.055240e−03	ko05111 Biofilm formation − Vibrio cholerae	Vancomycin

Table S2. Metagenomic KEGG pathways enriched in antibiotics groups, compared to the water control. Pathways shown have FDR corrected p-values < 0.01.
